# Serum and plasma levels of brain-derived neurotrophic factor in individuals with eating disorders (EDs): a systematic review and meta-analysis

**DOI:** 10.1186/s40337-022-00630-w

**Published:** 2022-07-18

**Authors:** Parnian Shobeiri, Sara Bagherieh, Parsa Mirzayi, Amirali Kalantari, Omid Mirmosayyeb, Antônio L. Teixeira, Nima Rezaei

**Affiliations:** 1 School of Medicine, Tehran University of Medical Sciences (TUMS), Children’s Medical Center Hospital, Dr. Qarib St., Keshavarz Blvd, Tehran, 14194 Iran; 2 Network of Immunity in Infection, Malignancy and Autoimmunity (NIIMA), Universal Scientific Education and Research Network (USERN), Tehran, Iran; 3 Non–Communicable Diseases Research Center, Endocrinology and Metabolism Population Sciences Institute, Tehran University of Medical Sciences, Tehran, Iran; 4 Research Center for Immunodeficiencies, Pediatrics Center of Excellence, Children’s Medical Center, Tehran University of Medical Sciences, Dr. Gharib St, Keshavarz Blvd, Tehran, Iran; 5 School of Medicine, Isfahan University of Medical Sciences, Esfahān, Iran; 6 Students’ Scientific Research Center (SSRC), Tehran University of Medical Sciences, Tehran, Iran; 7 Isfahan Neuroscience Research Center, Isfahan University of Medical Sciences, Esfahān, Iran; 8 Neuropsychiatry Program, Department of Psychiatry and Behavioral Sciences, McGovern Medical School, The University of Texas Health Science Center at Houston, Houston, TX USA; 9 Department of Immunology, School of Medicine, Tehran University of Medical Sciences, Tehran, Iran

**Keywords:** Brain-derived neurotrophic factor, BDNF, Eating disorder, Anorexia nervosa, Bulimia nervosa, Binge eating disorder

## Abstract

**Background:**

Brain-derived neurotrophic factor (BDNF) is essential for neuronal survival, differentiation, development, and plasticity. Evidence suggests that fluctuations in peripheral levels (i.e., plasma or serum) of BDNF are associated with eating behaviors. Nevertheless, the findings are inconsistent. The purpose of this study is to determine if serum or plasma levels of BDNF are altered in individuals with eating disorders (EDs) compared to controls.

**Methods:**

We conducted a systematic search of the core electronic medical databases from inception to March 2022 and identified observational studies that compared individuals with EDs to controls without EDs on serum or plasma levels of BDNF. R version 4.0.4 was used for all visualizations and calculations.

**Results:**

The current meta-analysis comprised 15 studies that fulfilled the inclusion criteria. Subjects with EDs (*n* = 795) showed lower BDNF levels compared to non-EDs controls (*n* = 552) (SMD: − 0.49, 95% CI [− 0.89; − 0.08], *p-value* = 0.01). Moreover, subgroup analysis was conducted based on the specimen (plasma and serum), which revealed no statistically significant difference in the levels of BDNF between the two subgroups (*p-value* = 0.92). Additionally, meta-regression results revealed that publication year, mean age of the individuals with EDs, NOS scores, and the number of individuals with EDs collectively accounted for 25.99% percent of the existing heterogeneity.

**Conclusion:**

Lower BDNF levels are associated with EDs.

**Supplementary Information:**

The online version contains supplementary material available at 10.1186/s40337-022-00630-w.

## Plain english summary

Brain-Derived Neurotrophic Factor, or BDNF for short, is one of the proteins in the body that regulates many functions of the brain, including how it develops and how its structure changes during its life. Neuropsychiatric disorders, including EDs, have been associated with changes in the amount of this protein in the body; but this is difficult to determine from one or two studies alone. In this review, we gathered information about the blood levels of BDNF in people suffering from ED and healthy people from several studies and compared them. This analysis showed that BDNF is lower in patients with ED. The significance of our review is that it might help understand the factors implicated in development of ED. It might also guide future studies to investigate decide whether BDNF can be used to predict ED relapse or remission.

## Background

Eating disorders (EDs) are a heterogeneous group of conditions defined by pathological eating habits that are linked with weight changes and/or social behaviors that have a major impact on a person's quality of life and ability to function socially [1, 2]. In this context, “disordered eating” refers to difficulty maintaining a healthy weight and/or body image, such as persistent dieting and weight concerns [3]. Concordantly, weight, diet, and negative body image concerns all contribute to the risk factors for ED [4]. Anorexia nervosa (AN), bulimia nervosa (BN), and binge eating disorder (BED) are three of the most common EDs with lifetime prevalence rates of 0.5–1%, 1–3%, and 2–2.5%, respectively [5].

Studies have shown that people at risk of developing EDs are also more likely to have co-occurring psychological issues such as anxiety, depression, and insomnia [3], possibly hinting to an overlap of pathology between these disorders. In addition, people with ED may develop serious somatic symptoms (e.g., pain, shortness of breath, fatigue or weakness), and report a decreased general well-being and quality of life [6], increasing their risk of suicide [7] and death rates [8, 9].

Brain-derived neurotrophic factor (BDNF) is a neurotrophin that plays a critical role in activity-dependent neuronal plasticity [10, 11] with a significant effect on neuronal morphology and physiology, enhancing neurite sprouting and synaptic stability, as well as long-term potentiation [11]. BDNF may be produced by different cell types in the body, including vascular structures, the immune system, neurons, and glial cells [12–14]. It can be measured in plasma or blood, and might reflect CNS levels of the molecule [15–17].

BDNF has been strongly implicated as one of the important regulators of eating behavior and its disturbances are associated with EDs [18, 19]. For instance, animal studies have shown that knockouts of BDNF induce hyperphagia and obesity [20], and BDNF administration decreases food intake, increases energy expenditure, and reduces body weight [21, 22]. Moreover, the hypothalamus and the dorsal vagal complex, two major autonomic centers believed to regulate eating behavior and energy balance, show high levels of expression of BDNF and its receptor [21].

Several studies have successfully linked polymorphisms of the BDNF gene to EDs [23–25]. Systematic reviews and meta-analyses have found positive correlations between decreased circulating BDNF levels and disordered eating [26, 27] and other psychiatric conditions [28]. Most of these studies are genetic studies, and those that analyze circulating levels of BDNF are either carried out in regard to one type of ED, or are outdated. In this context, this systematic review and meta-analysis aims to analyze the plasma or serum BDNF concentrations in individuals with different types of ED compared to controls according to the most recent literature, in order to explore the utility of BDNF measurements in diagnosis, classification, and prognosis of EDs. Possible associations between BDNF and the type of ED are also investigated.

## Material and methods

The current systematic review and meta-analysis was developed according to The Preferred Reporting Items for Systematic Reviews and Meta-Analyses (PRISMA 2020) guideline [29].

### Literature search and selection criteria

MEDLINE, Scopus, EMBASE, and Web of Science databases were searched online till May 16^th^, 2022, accordingly, to retrieve relevant investigations. In addition to searching electronic databases, we also verified the reference list of all pertinent publications already retrieved. Our search strategy is depicted in the supplementary material (Additional file 1). Titles and abstracts were screened independently by two authors (PM and AK), and a third reviewer (SB) resolved the conflicts in screening.

There were two criteria for inclusion: (1) A study was designed as an observational one, measuring BDNF levels in individuals with EDs (AN, BN, BED) without any other neurological or neuropsychiatric disorders; and (2) The article provided sufficient data, including the total number of subjects in both ED and controls, as well as mean and standard deviation (SD) of BDNF levels. Also, no restrictions for language were applied.

### Data extraction

The following data were extracted from each included article by one author (PM): (1) first author, year of publication, country of study, the assay used to measure BDNF levels, the type of specimen (serum, plasma, or blood), the number of subjects in the ED (e.g., AN, BN, BED) and control groups, the demographic characteristics of people with EDs and controls (e.g., age and gender), the mean and SD of BDNF levels in both the affected individuals and control groups, and the measurement scale used to calculate BDNF levels (e.g., pg./mL, ng/mL, or ng/mg).

### Quality assessment and risk of bias

The quality of included studies was assessed using the Newcastle–Ottawa scale (NOS), which was developed for observational studies. The NOS is constructed to evaluate three major features of observational studies: sample selection, case–control comparability, and exposure. Scores on this scale range from 0 to 9 [30]. Studies with a star rating of 7–9 were considered of the best quality, a rating of 4–6 stars a poor quality, and a rating of fewer than four the lowest quality [31]. Two authors (SB and PM) independently evaluated the quality of the included studies using the NOS. Different checklists were used based on the study design.

### Quantitative analysis

R version 4.0.4 (R Core Team [2020]. R: A language and environment for statistical computing. R Foundation for Statistical Computing, Vienna, Austria) was used for all calculations and visualizations. "meta" (version 4.17–0), "metafor" (version 2.4–0), "dmetar" (version 0.0–9), and "tidyverse" were utilized (version 1.3.0). R was used to create all forest and the drapery plots. Statistical significance was defined as a *p-value* of < 0.05.

The effect size was quantified using the standardized mean difference (SMD). The analytical model was composed of fixed effects and random effects, interchangeably. If the values reported in the manuscript were expressed as a median and interquartile range (IQR) or median and range and we were unable to obtain the mean and standard deviation (SD) from the authors, we converted these values using the statistical methods suggested by Luo et al. [32] and Wan et al. [33]. The Q statistic and the I^2^ index were used to determine heterogeneity. According to Cochrane standards, an I^2^ < 40% value indicates that inconsistency across studies is not significant. We intended to utilize the fixed effects model in this instance. If the I^2^ estimates varied by more than 40%, we expected to analyze using the random effects technique. To further elucidate the sources of heterogeneity, we performed a sensitivity analysis on meta-analyses with substantial heterogeneity, including ten or more papers. We removed one study each time and recalculated the effect size (Leave-One-Out Analyses).

The degree of asymmetry in the funnel plot and Egger's test [34] are used to identify publication bias. Indeed, funnel plots are often used to visually reveal publication bias. By contrast, the Egger's test is an objective statistic that enables users to validate visual cues provided by funnel plots. When there was evidence of publication bias, we used the trim-and-fill procedure to adjust the effect sizes [35].

## Results

### Selection of studies

**Fig. 1:**
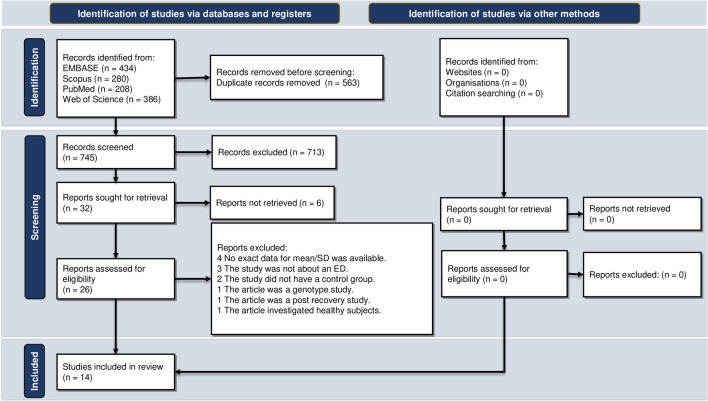
Flow diagram summarizing the selection of eligible studies based on the PRISMA guidelines

The search strategy retrieved a total yield of 1,308 studies. After removing the duplicates, 745 studies remained. The screening identified 32 potentially eligible studies, and 14 original studies met the criteria to be included in the meta-analysis. No further studies that were appropriate for inclusion were identified via hand searching and checking the references. Figure 1 illustrates the process of study selection according to the PRISMA guidelines.

### Study characteristics & major findings of the included studies

**Fig. 2:**
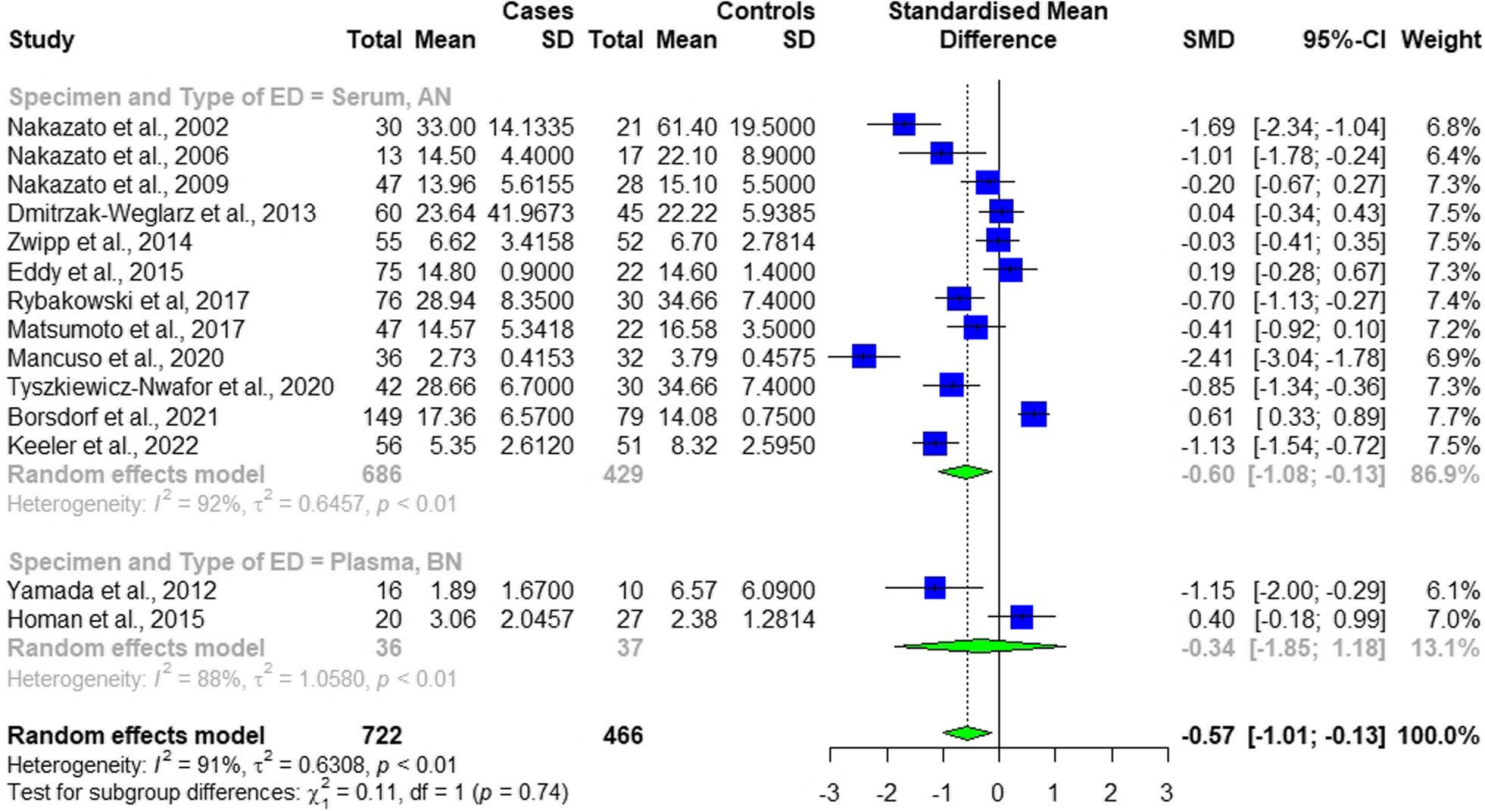
The forest plot of the subgroup analysis based on the specimen and type of ED, denoting a statistically significant difference in the level of BDNF between participants with and without eating disorder, but not between the two subgroups

Table 1 demonstrates the basic characteristics of the included studies in the meta-analysis. Levels of BDNF were measured in 14 studies from 2002 to 2022, all of which included control groups. 1,454 total observations, including 722 cases and 466 controls, were retrieved from the included studies. A total female predominancy was observed among the subjects with a 1188 to 0 female to male ratio. All the studies reported findings on one of the ED types, either Anorexia Nervosa (AN) or Bulimia Nervosa (BN). Regarding the four studies with longitudinal designs, the baseline levels of BDNF were analyzed [36–39]. The overall standardized mean difference (SMD) between ED individuals and the controls was − 0.49 [ − 0.0.9–0.08] (Fig. 2).

**Table 1:** Baseline Characteristics of Included Studies

Study ID					Patients			Controls		Main significant findings
Author, Year	Country	Study design	BDNF measurement method	Source (serum, plasma)	No. (Type of ED)	Diagnostic Criteria	BDNF levels (mean ± SD), ng/ml	No	BDNF levels (mean ± SD), ng/ml	
Nakazato et al., 2002	Japan	Cross-sectional	BDNF Emax Immunoassay System kit (Promega, Madison, WI)	Serum	12 (AN-R, AN-BP)	DSM-IV	24.9 ± 6.75	21	61.4 ± 19.5	Lower BDNF levels in AN and BN patients compared to controls Lower BDNF levels in AN patients compared to BN patients Positive correlation between BDNF levels and BMI in all subjects
Nakazato et al., 2002	Japan	Cross-sectional	BDNF Emax Immunoassay System kit (Promega, Madison, WI)	Serum	18 (BN)	DSM-IV	38.4 ± 15.3	21	61.4 ± 19.5	
Nakazato et al., 2006	Japan	Longitudinal	BDNF Emax Immunoassay System kit (Promega, Madison, WI, USA)	Serum	13 (AN-R, AN-BP)	DSM-IV	14.5 ± 4.4	17	14.5 ± 4.4	Lower BDNF levels in AN patients compared to controls No difference in BDNF levels in AN patients before and after partial weight recovery Positive correlation between BDNF levels and ED symptom scores (EDI-2) in all subjects Positive correlation between BDNF levels and BMI in all subjects
Nakazato et al., 2009	Japan	Cross-sectional	Emax Immunoassay System kit (Promega, Madison, WI, USA)	Serum	29 (AN-R, AN-BP)	DSM-IV	11.7 ± 4.9	28	15.1 ± 5.5	Lower BDNF levels in AN patients compared to controls Lower BDNF levels in AN patients compared to patients who recovered from AN Higher rate of set-shifting errors (WCST) in AN patients No correlation between BDNF levels and WCST performance
Yamada et al., 2012	Japan	Cross-sectional and longitudinal	ELISA kit (Quantikine, R & D Systems, Minneapolis, MN, USA)	Plasma	16 (BN)	DSM-IV	1.89 ± 1.67	10	6.57 ± 6.09	Lower BDNF levels in BN patients compared to controls Increased BDNF levels following inpatient treatment, suggesting that lower BDNF in BN is associated with abnormal eating behaviors, especially binge eating
Dmitrzak-Weglarz et al., 2013	Poland	Cross-sectional	DuoSet ELISA Development Kits (R&D Systems) –	Serum	46 (AN-R)	ICD-10 and DSM-IV	23.72955 ± 8.245963	45	22.22206 ± 5.938468	No difference in BDNF levels in AN patients compared with controls No correlation between BDNF levels and BMI or severity of depression symptoms (BDI) in all subjects Correlations between BDNF levels and patient personality dimensions (TCI)
Dmitrzak-Weglarz et al., 2013	Poland	Cross-sectional	DuoSet ELISA Development Kits (R&D Systems) –	Serum	14 (AN-BP)	ICD-10 and DSM-IV	23.32821 ± 8.078747	45	22.22206 ± 5.938468	
Zwipp et al., 2014	Germany	Cross-sectional and longitudinal	Enzyme-Linked Immunosorbent Assay kits (ELISA; 181 Promega Inc., Madison, WI, USA)	Serum	55 (AN)	DSM-IV	6.6165 ± 3.4158	52	6.7008 ± 2.7814	Higher BDNF levels in patients who recovered from AN compared to acutely underweight AN patients Increased BDNF levels with short-term weight gain in acutely underweight AN patients Inverse association of BDNF with psychomotor speed (TMT) in acutely underweight AN patients but not in controls Acutely underweight AN patients with higher BDNF also had lower lifetime BMI, indicating that serum BDNF levels in patients with AN vary with the stage of illness Changes in BDNF levels may have different context-dependent effects, including the modulation of cognitive functioning in acutely underweight patients; as BDNF has pleiotropic functions
Eddy et al., 2015	USA	Cross-sectional	immunoassay (ELISA, R&D Systems, Inc)	Serum	50 (AN-R)	DSM-V	13.6 ± 0.9	22	14.6 ± 0.8	Higher BDNF levels in AN-BP than AN-R No difference in BDNF levels in AN-BP compared to AN-R after controlling for BMI No difference in BDNF levels in AN patients compared to controls No correlation between BDNF levels and BMI in all subjects Positive association between BDNF levels and frequency of purging in all subjects
Eddy et al., 2015	USA	Cross-sectional	immunoassay (ELISA, R&D Systems, Inc)	Serum	25 (AN-BP)	DSM-V	17.2 ± 1.5	22	14.6 ± 1.4	
Homan et al., 2015	Switzerland	Randomized, double-blind, placebo-controlled, crossover study	BDNF Emax Immunoassay Kit (Promega, Switzerland)	Plasma	20 (Remitted BN)	DSM-IV	3.058 ± 1.5066	27	2.383 ± 1.2814	Positive correlation between AMPT–induced differences in BDNF levels with the AMPT–induced differences in reward learning across sample Higher BDNF levels in patients with remitted BN compared to controls across conditions Higher BDNF levels in the morning before compared with after a standardized breakfast across groups and conditions
Rybakowski et al., 2017	Poland	Longitudinal	n/a	Serum	76 (AN)	n/a	28.94 ± 7.19	30	34.66 ± 7.4	Lower BDNF levels in acute AN Normalization of BDNF levels after weight recovery in AN Negative correlation between BDNF levels and body weight in acute AN but not AN after partial weight recovery No correlation between BDNF levels and psychopathological symptoms (HDRS, BDI and YBOCS) in either acute AN or AN after partial weight recovery No evidence found to support a role of serum BDNF levels in the modulation of depressive and obsessive–compulsive symptoms of AN
Matsumoto et al., 2017	Japan	Cross-sectional	Human BDNF ELISA kits (Aviscera Bioscience, Santa Clara, CA, USA)	Serum	19 (AN-R, AN-BP)	DSM-IV	13.96 ± 6.02	22	16.58 ± 3.5	No difference in proBDNF and mBDNF levels among AN patients, BN patients, and controls Correlation between proBDNF and MMP-9 levels in both ED patients and controls Positive correlation between mBDNF levels and IGT performance in BN patients but not in AN patients
Matsumoto et al., 2017	Japan	Cross-sectional	Human BDNF ELISA kits (Aviscera Bioscience, Santa Clara, CA, USA)	Serum	28 (BN)	DSM-IV	14.99 ± 4.9	22	12.57 ± 12.54	
Mancuso et al., 2020	USA	Cross-sectional	ELISA (EMD Millipore: Billerica, MA)	Serum	36 (AN-R, AN-BP)	K-SADS PL and EDE	2.7313 ± 0.4153	32	3.7928 ± 0.4575	Lower fasting BDNF levels in AN patients compared with controls Lower BDNF AUC after breakfast in AN patients compared with controls Positive association between BDNF AUC and kilocalories consumed during CTT in AN patients (particularly AN-R)
Tyszkiewicz-Nwafor et al., 2020	Poland	Longitudinal	BDNF DuoSet (cat. No DY 248) and TrkB DuoSet (cat. No DY 397–5) ELISA Development Kit (R&D System, Minneapolis, MN, USA)	Serum	42 (AN)	ICD-10, DSM-IV and DSM-V	28.66 ± 6.7	30	34.66 ± 7.4	Increased BDNF levels in AN patients after partial weight recovery compared with before, but lower levels compared with controls at two-time points Negative correlation between BDNF levels and the severity of ED symptoms (EAT-26) No correlation between BDNF levels and depressive and obsessive–compulsive symptoms (HDRS, BDI, YBOCS) for either malnourished patients or partially weight recovered AN patients
Borsdorf et al., 2021	Germany	Longitudinal	Quantikine ELISA (R&D Systems Inc.)	Serum	149 (AN)	DSM-IV	17.36 ± 6.57	79	14.08 ± 0.75	Lower BDNF levels in AN patients at admission compared with controls Continuous increase in BDNF levels, reaching supranormal levels at 2.5-year follow-up Inverse association between BDNF levels with ED psychopathology (BDI-II, SCAS, MROAS, SIAB-EX) at discharge Positive association between BDNF levels with previous weight gain at 1-year follow-up
Keeler et al., 2022	UK	Cross-sectional	U-PLEX Human BDNF assay (Meso Scale Discovery, Maryland, USA)	Serum	56 (AN-R, AN-BP)	DSM-V	5.354 ± 2.616	51	8.323 ± 2.595	Lower BDNF levels in AN patients compared to controls and patients recovering from AN Negative association of BDNF with depression and ED psychopathology in the whole sample, but not in AN patients BDNF serum concentrations may be a state marker of AN, but do not reflect symptom severity In acute AN, BDNF levels seem to be linked to TNF-α signaling

*BDNF*: Brain-Derived Neurotrophic Factor; *proBDNF*: precursor Brain-Derived Neurotrophic Factor; *mBDNF*: mature Brain-Derived Neurotrophic Factor; *ED*: Eating Disorder; *AN*: Anorexia Nervosa; *BN*: Bulimia Nervosa; *AN-R*: Anorexia Nervosa Restricting subtype; *AN-BP*: Anorexia Nervosa Binge-eating/Purging subtype; *YFAS:* Yale Food Addiction Scale; *ELISA*: Enzyme-Linked Immunosorbent Assay; *BMI*: Body Mass Index; *DSM*: Diagnostic and Statistical Manual of Mental Disorders; *ICD*: International Classification of Diseases; *K-SADS PL*: Schedule for Affective Disorders and Schizophrenia for School Age Children-Present and Lifetime Version; *EDE*: Eating Disorder Examination; *HDRS*: Hamilton Depression Rating Scale; *BITE*: Bulimic Investigatory Test, Edinburgh; *EDI*: Eating Disorder Inventory; *WCST*: Wisconsin Card Sorting Test; *TCI*: Temperament and Character Inventory; *TMT*: Trail Making Test; *AMPA*: Alpha-methyl-para-tyrosine; *MMP*: Matrix Metalloproteinase; *IGT*: Iowa Gambling Task; *SNP*: Single Nucleotide Polymorphism; *AUC*: Area Under Curve; CTT: Cookie Taste Test; *EAT*: Eating Attitudes Test; *BDI*: Beck Depression Inventory; *YBOCS*: Yale-Brown Obsessive–Compulsive Scale; *SCAS*: Spence Children’s Anxiety Scale; *MROAS*: Morgan–Russell Outcome Assessment Schedule; *SIAB-EX*: Structured Interview for Anorexia and Bulimia-Expert Interview

Except for four studies [37, 40–42], all the other included ones reported a negative association between the level of BDNF and the presence of any type of eating disorders, meaning the levels of BDNF was lower among ED individuals compared to healthy controls (Table 1). None of the four studies reported the opposite recruited sex-matched controls. In fact, except for Borsdorf et al. [37] study, having age-matched controls, the other three studies did not provide information on the detailed characteristics of their control groups.

### The methodological quality of studies

In the present study, nine studies achieved above 6 stars, six studies achieved 6 stars, and one study achieved 5 stars (Table 2). The median NOS score for included studies was 6.57 (IQR = 1, mean ± SD = 6.57 ± 0.75, range: 5–8) out of 9, which shows an estimated moderate to good quality. One study (7.14%) [43] had high risks of bias (scores 0–5), twelve studies (85.71%) [36, 38–42, 44–48] had moderate [34, 39] risks of bias (scores 6–7), and one study (7.14%) [37] had low risks of bias (scores 8–9) in their methodological quality (Table 2).

**Table 2:** Quality assessment of the included studies based on the NOS checklist** (**adapted for cross sectional studies**)**

Author, Year	QUESTION 1	QUESTION 2	QUESTION 3	QUESTION 4	QUESTION 5	QUESTION 6	QUESTION 7	Overall
Nakazato et al., 2002	*	*	*	**		*	*	**7**
Nakazato et al., 2006		*	*	**	*	*		**6**
Nakazato et al., 2009	*	*	*	**	*		*	**7**
Yamada et al., 2012	*		*		*	*	*	**5**
Dmitrzak-Weglarz et al., 2013	*	*	*	**	*	*		**7**
Zwipp et al., 2014	*	*		*	*	*	*	**6**
Eddy et al., 2015	*	*	*		**	*	*	**7**
Homan et al., 2015	*		*	**	*		*	**6**
Rybakowski et al., 2017	*	*	*	*	**		*	**7**
Matsumoto et al., 2017	*	*	*	**		*	*	**7**
Mancuso et al., 2020	*	*	*	**	*		*	**7**
Tyszkiewicz-Nwafor et al., 2020	*	*	*	*	*		*	**6**
Borsdorf et al., 2021	*	*	*	*	**	*	*	**8**
Keeler et al., 2022	*	*		*	**		*	**6**

*Selection*: Questions 1 to 4, *Comparability*: Question 5, *Outcome:* Questions 6 and 7

*, ** The NOS checklist uses a "star system" to assess the quality of studies, with the number of stars indicating the overall score in that particular domain, i.e., Selection, Comparability, and Outcome

### Comparison of BDNF Levels between Individuals with EDs and Controls

In all studies, levels of BDNF were compared between individuals with EDs (*N* = 722) and controls (*N* = 466). The cumulative number of female and male participants was 1188 and 0, respectively. The mean age was 19.99 ± 6.8 and 22.15 ± 8.48 for participants with EDs and controls, respectively.

A statistically significant difference was observed comparing the levels of BDNF between participants with EDs and controls (SMD − 0.5667, 95% CI [ − 1.0067; − 0.1267], *p-value* = 0.0116, Fig. 2). Two sets of subgroup analysis were conducted:

1. First, based on the specimen (plasma and serum), which revealed that there was not a statistically significant difference in the levels of BDNF between the two subgroups (*p-value* = 0.7425, Fig. 2).
2. Second, based on ED type. As such, studies were categorized into two subgroups based on the type of EDs that studies’ sample size had: Bulimia Nervosa (BN) and Anorexia Nervosa (AN), and Obesity Associated with ED. This analysis demonstrated that there was not a statistically significant difference between the two subgroups (*p-value* = 0.7425, Fig. 2).

### Between-study heterogeneity

**Fig. 3:**
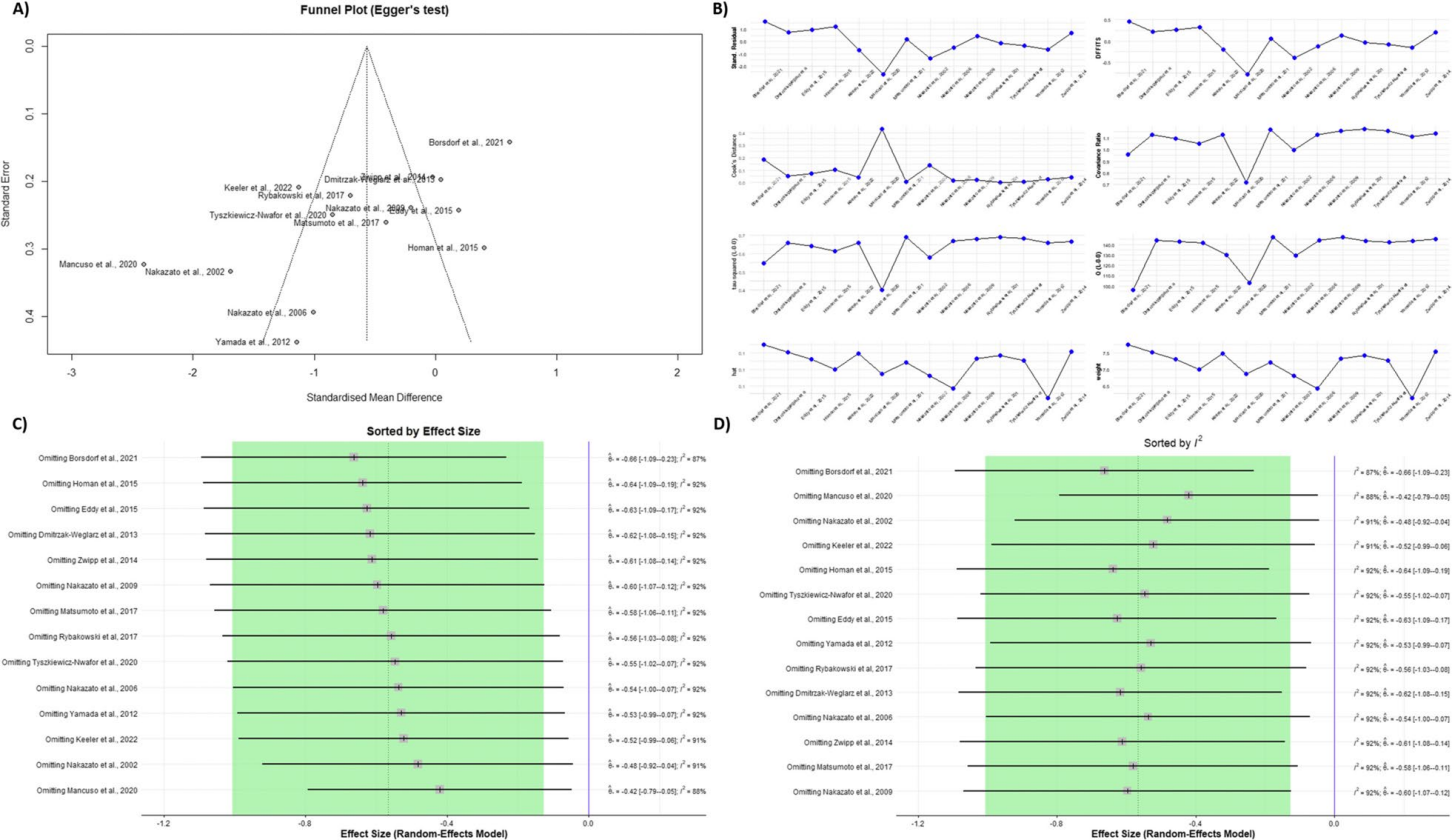
**A** The funnel plot showing no evidence of publication bias, statistically supported by Egger’s regression test. **B** The influence analysis plot, showing different influence diagnostics including: Externally Standardized Residuals, DFFITS Value, Cook’s Distance, Covariance Ratio, Leave-One-Out τ2 and Q Values, and Hat Value and Study Weight. **C**eave-One-Out sensitivity analysis result, sorted by effect sizes from low to high. **D** Leave-One-Out sensitivity analysis result, sorted by heterogeneity as measured by I2 from low to high

The Eggers’ test did not indicate the presence of substantial funnel plot asymmetry (*p-value* = 0.0100, Fig. 3A). The between-study heterogeneity was statistically significant (*p-value* =  < 0.0001). Its variance was estimated at τ^2^ = 0.6308 [0.2969; 1.7793], with an I^2^ value of 91.2% [86.9%; 94.0%]. The prediction interval ranged from g = − 2.3650 to 1.2317, indicating that negative intervention effects cannot be ruled out for future studies (Fig. 3B).

### Outliers’ identification and sensitivity analysis

An influence analysis was done to detect any influential cases, which identified three studies as outliers [37, 44, 46], and the *p-value* raised from 0. 0116 to 0.0118. Consequently, we conducted a sensitivity analysis to identify whether the influential cases have affected the significancy level of the meta-analyses. To do so, each time, we omitted one study and recalculated the effect size (leave-one-out analysis). Sensitivity analysis showed that the effect size remained significant after omitting each study (Fig. 3C, D).

### Univariate and multivariate meta-regression analysis

To identify the source of heterogeneity, a meta-regression analysis was conducted. Number of individuals with EDs, the year of publication, and the mean age of ED individuals accounted for 24.56, 49.99, and 84.39 percent of heterogeneity, respectively. On the other hand, the NOS scores of the included studies did not play a part in the observed heterogeneity of the data per se. A multivariate meta-regression analysis was done to rule out the existence of any possible overlaps between the number of individuals with EDs and the other three mentioned variables that did not contribute as much to the heterogeneity, revealing publication year and the number of individuals with EDs contributed to 34.37 percent of the heterogeneity, NOS scores and number of individuals with EDs contributed to 42.58 percent of the heterogeneity, and mean age of individuals with EDs and their sample size contributed to 51.41 percent of the heterogeneity. Finally, all the four variables including publication year, mean age of the individuals with EDs, NOS scores, and the number of individuals with EDs collectively accounted for 66.8 percent of heterogeneity in the data (Table 3).

**Table 3:** Meta − regression of BDNF levels in persons with EDs and healthy controls

Moderator	No. of comparisons	No. of subjects		Meta-regression				R^2^ analog (proportion of variance explained)
		ED	HC	Estimated Intercept	95% CI		*p*-value	
No. of persons with EDs	14	722	466	− 1.2303	− 1.9597	− 0.5009	0.0331	24.56%
Age (mean, years)	14	722	466	0.6128	− 0.0624	1.2880	< 0.0001	84.39%
Publication year	14	722	466	− 1.6911	− 2.9995	− 0.3826	0.0013	46.99%
NOS score	14	NA	NA	− 1.1455	− 2.9337	0.6426	0.4344	0.00%
All moderators combined (Multiple Meta − Regression)	13	NA	NA	51.8716	− 157.1692	260.9124	0.1098	66.80%

## Discussion

To the best of our knowledge, the current study is the first systematic review and meta-analysis comparing the levels of BDNF in people with eating disorders (EDs) to people without a history of EDs. The main finding of our study is that BDNF levels are significantly lower among individuals with EDs compared to healthy controls. Previous systematic reviews assessed the levels of BDNF in individuals with AN [24, 27, 47] and BN [26] separately, showing that levels of BDNF are lower among affected individuals. Given the dynamic nature of EDs, our study takes a more comprehensive approach and compares: First, persons with EDs of any kind to healthy controls; Second, the discrepancies in the levels of BDNF between different types of ED.

Our findings demonstrate that people with EDs generally have a lower level of serum and plasma BDNF compared to healthy controls. Several hypotheses can be proposed to explain lower BDNF blood levels in people with EDs. Since BDNF plays a crucial role in the nervous system's growth, regulation, and maintenance, its level alterations can affect neurological functions, including hunger and satiety, both directly and via indirect pathways [49, 50]. Both of these pathways can eventually lead to disturbances in eating habits and give rise to respective disorders. Previous research has provided a large amount of evidence emphasizing BDNF’s control of feeding, with specific attention to the central effects [51]. Nevertheless, BDNF levels might also reflect other pathophysiological mechanisms associated with EDs and be secondary to other underlying factors.

Regarding the direct pathway by which decreased BDNF levels mediate disruption of eating habits, it has been suggested that single nucleotide polymorphisms (SNPs) in the BDNF gene increase the susceptibility to the development of binge eating disorder, and people with EDs have an amplified level of genetically-altered BDNF molecules. Such functional polymorphisms are often associated with lower blood levels of BDNF. Due to the high prevalence of this specific mutation, it has been more examined than other BDNF gene SNPs, specifically regarding weight regain. In a study conducted by Nonino et al., the detection of allele frequency of *rs6265* SNP in the BDNF gene indicated an elevated risk for the development of BED in individuals with relapse to obesity in the postoperative period of bariatric surgery [52]. Not only is it presumed that an association between the frequency of SNPs within the BDNF gene and the risk of ED onset exists, but it has also been suggested that such polymorphisms can contribute to episodes of EDs exacerbations in people already diagnosed with EDs. Montelenoe et al. have also demonstrated that the *rs6365* polymorphism of the BDNF gene is significantly associated with binge eating behavior in women with bulimia nervosa or binge eating disorder [53]. This is particularly important in designing more efficient treatment plans to battle ED.

BDNF levels can alter neuronal function in an indirect manner as well. A growing body of evidence suggests that BDNF is involved in depression, as its levels are significantly reduced in depressed individuals [54]. As such, lower BDNF levels raises the vulnerability to depression, with which eating disorder frequently co-exist as they share common biological mechanisms [55, 56]. Furthermore, it has been shown that antidepressant agents up-regulate the expression of the BDNF gene [55, 57]. Therefore, the assumption that dysregulated levels of BDNF can indirectly, using depression as a mediator, put individuals at higher risk for developing EDs is further confirmed.

Recent findings have also provided evidence in favor of the interplay between BDNF, insulin, insulin’s counter-regulatory hormones, including catecholamines, and leptin levels in people with EDs [58]. They suggest that leptin and BDNF levels are sensitive to the depletion of catecholamine reserve. Under physiological circumstances, exhaustion of catecholamine stores in the body, such as the pre-prandial situations, causes the BDNF levels to fall drastically and then surge again once the individual is in a postprandial state inducing appetite suppression [42]. In people with EDs, the above mechanism loses sensitivity to catecholamine depletion and shows more preference towards the pre-prandial states, leading to significantly lower levels of BDNF, which can, in turn, initiate the downstream cascade of pathological dysregulations observed in ED conditions.

As mentioned earlier in the discussion, not only do disrupted BDNF levels give rise to the occurrence of EDs, they can impact the severity of their symptoms [36]. Additionally, recent studies suggest that on a hypothetical spectrum of disordered eating behaviors, individuals with more extreme symptoms had significantly lower BDNF levels as well [18, 41]. Further research is required to determine whether there is a causal relation between BDNF levels and the severity of symptoms or whether they are both secondary to another underlying factor contributing to the disorder's pathophysiology.

One interesting aspect of the present study is that it suggests persons with EDs, regardless of their exact type, do not differ much in terms of BDNF levels. This is contrary to the findings of some of the included studies and may, in part, be due to the larger sample sizes of those included studies whose findings were in line with the above argument.

The present study has some strengths and limitations that merit comment. It is the first meta-analysis comparing the level of BDNF in individuals across the eating disorder spectrum to healthy controls. It sheds some light on the importance of BDNF as a member of the neurotrophin family of growth factors as an underlying attributable risk factor for eating disorders. However, there are some limitations, too. Further research is required to estimate the prevalence of depression and BDNF levels simultaneously to depict a more detailed picture of the interplay between depression and BDNF levels in the context of eating disorders, and to reach a clear understanding of the delicate interactions between BDNF levels and the entire nervous system to unravel the full scope of physiological, biochemical, and even anatomical influences this factor has on hunger regulation. Also, due to the lack of information in included studies, we were unable to subgroup the findings based on the applied diagnostic methods, which could lead to more precise results or even illuminate possible sources of bias. Another notable limitation could be the fact that all the individuals in the included studies are female. This hinders our ability to reach generalizable findings and also to perform a meta-regression analysis with sex as one of the variables since it has only of class of item.

## Conclusion

This study showed that lower levels of blood BDNF are associated with the presence of EDs. Whether or not a causative relationship exists between the two phenomena is a question for further research to address. Considering the burden of EDs mentioned in previous studies and the possible role of altered BDNF levels in the onset and progression of EDs, interventions targeting the restoration of BDNF level to normal are deemed beneficial in the affected people.

## Supplementary Information

**Additional file 1.** Summary of search strategies customized for each data bank.

## Data Availability

Not applicable.

## Abbreviations

AN: Anorexia nervosa
BDNF: Brain-derived neurotrophic factor
BED: Binge eating disorder
BN: Bulimia nervosa
CNS: Central nervous system
EDs: Eating disorders
NOS: Newcastle–Ottawa scale
RAN: Restricting type anorexia nervosa
SNPs: Single nucleotide polymorphisms
