# Supplementary material for: Serum and plasma levels of brain-derived neurotrophic factor in individuals with eating disorders (EDs): a systematic review and meta-analysis
Source: J Eat Disord. 2022 Jul 18;10:105. doi: 10.1186/s40337-022-00630-w (PMC9295529; doi:10.1186/s40337-022-00630-w)
Supplement: Supplementary file 1 — Additional file 1. Summary of search strategies customized for each data bank. [file 40337_2022_630_MOESM1_ESM.docx]

**Supplementary material**

**Title: Serum and Plasma Levels of Brain-Derived Neurotrophic Factor in Individuals with Eating Disorders (EDs): A Systematic Review and Meta-Analysis**

**Authors:** Parnian Shobeiri, Sara Bagherieh, Parsa Mirzayi, Amirali Kalantari, Omid Mirmosayyeb, Nima Rezaei*

***Corresponding author:**

- Nima Rezaei, MD, Ph.D., Research Center for Immunodeficiencies, Children’s Medical Center, Dr. Gharib St, Keshavarz Blvd, Tehran, Iran, E-mail: [rezaei_nima@yahoo.com](mailto:rezaei_nima@yahoo.com)

***Table of Contents***

PubMed2

EMBASE2

Scopus2

Web of Science2

1. **Search Strategies**
   1. **PubMed**

| #1 | ((Brain-Derived Neurotrophic Factor[MeSH Major Topic]) OR (Brain-Derived Neurotrophic Factor[MeSH Terms]) OR (Brain-Derived Neurotrophic Factor) OR (BDNF*)) AND ((Feeding and Eating Disorders[MeSH Major Topic]) OR (Feeding and Eating Disorders[MeSH Terms]) OR Eating Disorder* OR Anorexia* OR Bulimia*) | 203 |
| --- | --- | --- |

- 1. **EMBASE**

| #1 | ('eating disorder'/exp OR 'ednos' OR 'osfed' OR 'disordered eating' OR 'disordered eating behavior' OR 'disordered eating behaviour' OR 'eating disorder' OR 'eating disorders' OR 'eating pathology' OR 'feeding and eating disorder' OR 'feeding and eating disorders' OR 'pathologic eating behavior' OR 'pathologic eating behaviour' OR 'pathologic feeding behavior' OR 'pathologic feeding behaviour' OR 'pathological eating' OR 'pathological feeding behavior' OR 'pathological feeding behaviour' OR 'pathological feeding condition' OR 'pathological feeding disorder' OR 'bulimia'/exp OR 'bulimia' OR 'bulimia nervosa' OR 'diabulimia' OR 'hyperrexia' OR 'nervous bulimia' OR 'anorexia'/exp OR 'anorexia' OR 'anorexic') AND ('brain derived neurotrophic factor'/exp OR 'bdnf' OR 'brain derived neurotrophic factor' OR 'brain-derived neurotrophic factor') | 431 |
| --- | --- | --- |

- 1. **Scopus**

| #1 | TITLE-ABS-KEY(bdnf OR Brain-Derived Neurotrophic Factor OR Brain Derived Neurotrophic Factor) AND TITLE-ABS-KEY(Eating Disorder* OR Feeding Disorder* OR Anorexia* OR Bulimia*) | 276 |
| --- | --- | --- |

- 1. **Web of Science**

| #1 | (BDNF OR Brain-Derived Neurotrophic Factor) AND (Eating Disorder* OR Feeding Disorder* OR Anorexia* OR Bulimia*) | 381 |
| --- | --- | --- |
